# Differential Roles of Mitochondrial Translocation of Active Caspase-3 and HMGB1 in Neuronal Death Induced by Status Epilepticus

**DOI:** 10.3389/fncel.2018.00301

**Published:** 2018-09-05

**Authors:** Ji-Eun Kim, Tae-Cheon Kang

**Affiliations:** Department of Anatomy and Neurobiology, Institute of Epilepsy Research, College of Medicine, Hallym University, Chuncheon, South Korea

**Keywords:** epilepsy, Mdivi-1, mitochondrial fission, mitochondrial fusion, parvalbumin, seizure, WY14643

## Abstract

Under pathophysiological conditions, aberrant mitochondrial dynamics lead to the different types of neuronal death: excessive mitochondrial fission provokes apoptosis and abnormal mitochondrial elongation induces necrosis. However, the underlying mechanisms how the different mitochondrial dynamics result in the distinct neuronal death patterns have been elusive. In the present study, status epilepticus (SE) evoked excessive mitochondrial fission in parvalbumin (PV) cells (one of GABAergic interneurons) and abnormal mitochondrial elongation in CA1 neurons in the rat hippocampus. These impaired mitochondrial dynamics were accompanied by mitochondrial translocations of active caspase-3 and high mobility group box 1 (HMGB1) in PV cells and CA1 neurons, respectively. WY14643 (an activator of mitochondrial fission) aggravated SE-induced PV cell loss by enhancing active caspase-3 induction and its mitochondrial translocation, which were attenuated by Mdivi-1 (an inhibitor of mitochondrial fission). Mitochondrial HMGB1 import was not observed in PV cell. In contrast to PV cells, Mdivi-1 deteriorated SE-induced CA1 neuronal death concomitant with mitochondrial HMGB1 translocation, which was abrogated by WY14643. These findings suggest that SE-induced aberrant mitochondrial dynamics may be involved in translocation of active caspase-3 and HMGB1 into mitochondria, which regulate neuronal apoptosis and necrosis, respectively.

## Introduction

Mitochondria play an essential role in bioenergetics and respiratory functions for cell viability. Furthermore, mitochondria are dynamic organelles, which continuously change their morphologies (referred to as dynamics) to maintain their functionality in response to extra- and intracellular circumstances by two counteracting processes of fusion (elongation) and fission (fragmentation). Furthermore, imbalance of mitochondrial dynamics lead to the distinct neuronal death under stressful conditions (Youle and Karbowski, 2005; Parone et al., 2008; DuBoff et al., 2012; Kim et al., 2014; Kim and Kang, 2017): Excessive mitochondrial fission provokes the impaired mitochondrial function triggering apoptosis. In contrast, improper segregations and localizations of mitochondria induced by aberrant mitochondrial elongation lead to necrosis (Parone et al., 2008; DuBoff et al., 2012; Kageyama et al., 2012).

In the hippocampus, dentate hilar neurons and CA1–3 pyramidal cells are extremely vulnerable to status epilepticus (SE, a prolonged seizure activity), although dentate granule cells are resistant. Furthermore, SE-induced neuronal death shows the heterogeneous patterns (Ordy et al., 1993; Mathern et al., 1995; Wittner et al., 2001; Kim et al., 2011; Ryu et al., 2011). Briefly, SE leads to programed necrosis in CA1 neurons (Kim et al., 2014; Ko et al., 2015; Hyun et al., 2016), and caspase-3 dependent apoptosis in loss of parvalbumin (PV) cell (one of GABAergic fast-firing interneurons in the dentate gyrus; Kang et al., 2006; Kim and Kang, 2017). Interestingly, the properties of mitochondrial dynamics in response to SE are also distinct from each neuronal subpopulation. SE provokes aberrant mitochondrial elongation in CA1 neurons (Kim et al., 2014; Hyun et al., 2016), but it propels excessive mitochondrial fragmentation in PV cell (Kim and Kang, 2017). However, the underlying mechanisms how the different mitochondrial dynamics lead to the distinct neuronal death patterns have been elusive.

High mobility group box 1 (HMGB1, a non-histone DNA-binding protein), is immediately released from the nucleus to the cytoplasm undergoing necrosis and involved in giant mitochondrial formation (Scaffidi et al., 2002; Faraco et al., 2007; Gdynia et al., 2010). Furthermore, mitochondrial HMGB1 transport facilitates and deteriorates programed necrotic CA1 neuronal death induced by SE (Hyun et al., 2016). Similar to HMGB1, active (cleaved) caspase-3 translocate from cytosol to mitochondria and disintegrates mitochondrial functions during apoptosis (Chandra and Tang, 2003). Therefore, pertinent questions are raised whether mitochondrial dynamics influence mitochondrial translocations of active caspase-3 and HMGB1, and whether their mitochondrial translocations are practically involved in the distinct neuronal death induced by SE.

Here, we demonstrate that under physiological condition mitochondrial fission was required for caspase-3 activation. Subsequently, active caspase-3 penetrated into mitochondria. Following SE, mitochondrial translocations of active caspase-3 were restricted to PV cells, and facilitated PV cell loss. However, HMGB1 preferentially permeated into elongated mitochondria in CA1 neurons following SE. Therefore, our findings suggest that mitochondrial imports of active caspase-3 and HMGB1 may be involved in the abnormal mitochondrial machinery-mediated neuronal death induced by SE.

## Materials and Methods

### Experimental Animals and Chemicals

Male Sprague–Dawley (SD) rats (7 weeks old) were used in the present study. Animals were kept under controlled environmental conditions (23–25°C, 12 h light/dark cycle). Rats freely accessed to water and standard laboratory food during the experiment. All animal protocols were approved by the Administrative Panel on Laboratory Animal Care of Hallym University (the authorization number of the IACUC, Hallym 2018-2). All possible efforts were taken to avoid animals’ suffering and to minimize the number of animals used during the experiment. All reagents were obtained from Sigma-Aldrich (St. Louis, MO, USA), except as noted.

### Intracerebroventricular Infusion

Under Isoflurane anesthesia (3% induction, 1.5%–2% for surgery and 1.5% maintenance in a 65:35 mixture of N_2_O:O_2_), animals were implanted a brain infusion kit 1 (Alzet, Cupertino, CA, USA) into the right lateral ventricle on the stereotaxic frame (1 mm posterior; 1.5 mm lateral; 3.5 mm depth to the Bregma). The infusion kit was sealed with dental cement and connected to an osmotic pump (1007D, Alzet, Cupertino, CA, USA). Each osmotic pump contained: (1) vehicle; (2) Mdivi-1 (50 μM) or (3) WY14643 (150 μM). An osmotic pump was placed in a subcutaneous pocket between scapulas. In our previous studies (Kim et al., 2014; Kim and Kang, 2017), the concentration of each compound could not affect the seizure activity in response to pilocarpine.

### SE Induction

Three days after surgery, LiCl (3 mEq/kg, i.p.) was administrated to all rats 24 h prior to pilocarpine hydrochloride treatment (30 mg/kg, i.p.). Twenty minutes before pilocarpine or saline (control), rats were given atropine methylbromide (5 mg/kg, i.p.) to inhibit peripheral effects of pilocarpine. Two hours after SE onset, diazepam (Valium; Hoffman la Roche, Neuilly sur-Seine, France; 10 mg/kg, i.p.) was administered and repeated, as needed.

### Tissue Processing

At the designated time points (control, 6 h, 12 h and 3 days after SE induction), animals were anesthetized with urethane (1.5 g/kg, i.p.), and transcardially perfused with phosphate-buffered saline (PBS) followed by 4% paraformaldehyde (in 0.1 M phosphate buffer; pH 7.4). The brains were removed and post-fixed by the same solution for 4 h at 4°C, and submerged overnight in 30% sucrose in 0.1 M PBS for cryoprotection. The 30 μm-thick coronal sections were made with a cryostat, and contained in six-well plates containing PBS. During sections, we confirmed the intracerebroventricular location of a brain infusion kit. Only animals showing the exact position were used in the present study.

### Immunohistochemistry and Fluoro-Jade B Staining

After incubation with 10% normal goat serum (Vector, Burlingame, CA, USA), section were reacted in the mixture of primary antibodies listed in Table 1 (in PBS containing 0.3% triton X-100) at room temperature for overnight. After washing, sections were incubated for 1 h in a FITC (green)-, Cy3 (red)- or AMCA (blue)-conjugated secondary antibodies (Vector, Burlingame, CA, USA). For negative control, tissues were reacted with pre-immune serum instead of primary antibody. Negative control tissues did not show any immunoreactivity for primary antibody (data not shown). To analyze the neuronal damage, Fluoro-Jade B (FJB) staining was performed according to the manufacturer’s instructions. Immunoreactivities were observed using an AxioImage M2 microscope or a confocal laser scanning microscope (LSM 710, Carl Zeiss Inc., Oberkochen, Germany).

**Table 1:** Primary antibodies used in the present study.

Antigen	Host	Manufacturer (catalog number)	Dilution used
Mitochondrial marker (Mitochondrial complex IV subunit 1, MTCO1)	Mouse	Abcam (#ab14705)	1:500
Active (cleaved) caspase-3	Rabbit	Cell signaling (#9664)	1:400
HMGB1	Rabbit	Abcam (#ab18256)	1:100
PV	Goat	Swant (#PVG213)	1:100,000

### Cell Count and Measurement of Mitochondrial Length and Mitochondrial HMGB1 and Active Caspase-3 Intensities

Areas of interest (1 × 10^5^ μm^2^) were selected in the captured images of the dentate gyrus and the CA1 region of the hippocampus proper (15 sections per each animal), and cell numbers were counted. The number of FJB-positive neurons was also counted by the same methods. For measurement of mitochondrial length, 25 serial images (z-stack, 1 μm) were obtained from each hippocampal section and were used for 3D-reconstruction by using ZEN lite software (Blue Edition, Carl Zeiss Inc., Oberkochen, Germany). Thereafter, we measured individual mitochondrion length (long-axis) and intensities of mitochondrial HMGB1 and active caspase-3 in PV cells and CA1 neurons (*n* = 20/section; Ko et al., 2016; Kim and Kang, 2017).

### Quantification of Data and Statistical Analysis

Student’s *t*-test or ANOVA were used to analyze statistical significance. Bonferroni’s test was applied for *post hoc* comparisons. A *p*-value below 0.05 was considered statistically significant.

## Results

### SE-Induced Mitochondrial Caspase-3 Transport in PV Cell, Not CA1 Neurons

**Figure 1:**
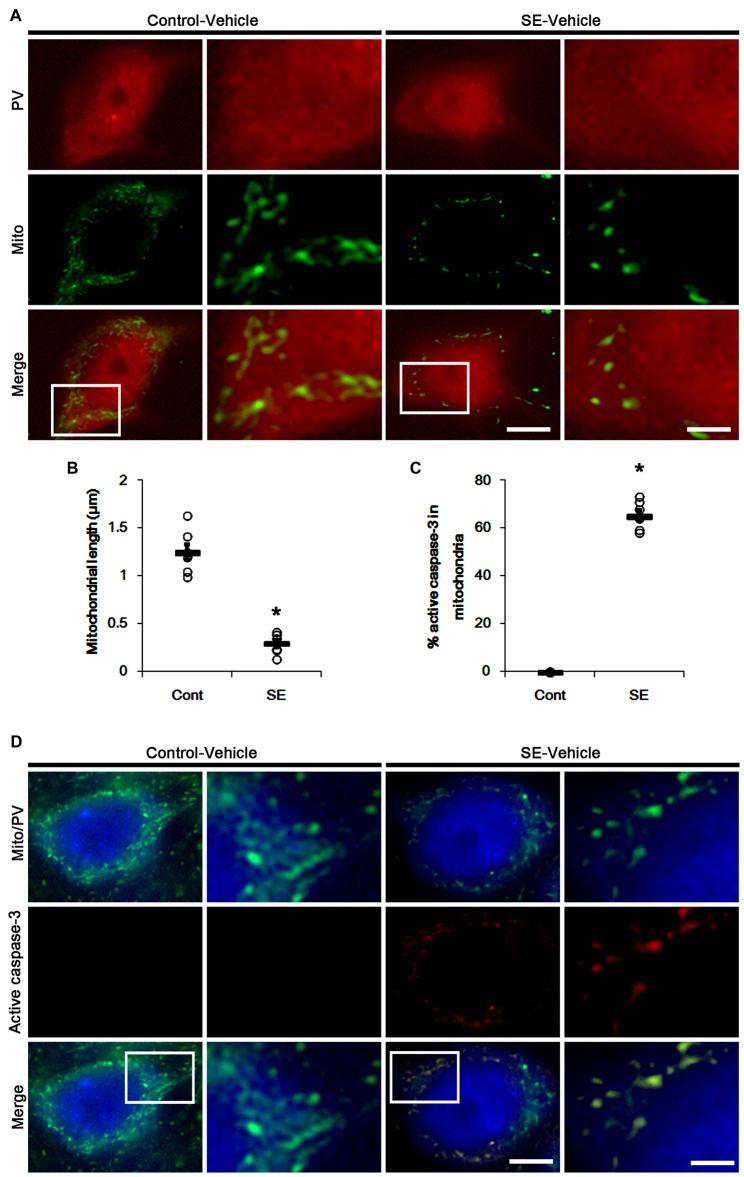
Excessive mitochondrial fission and active caspase-3 induction in parvalbumin (PV) cells 6 h after status epilepticus (SE). SE rapidly induces mitochondrial fragmentation and translocation of active capase-3 to mitochondria in PV cells of vehicle-treated animals. **(A)** Representative photos of mitochondria (Mito, green) in PV (red) cells. Rectangles in merge panels (left) indicate the zoom areas for the high magnification photos (right). Bar = 5 (left panels) and 1.5 (right panels) μm. **(B,C)** Quantification of the mitochondrial length in PV cells **(B)** and the fraction of active caspase-3 positive mitochondria in total mitochondria **(C)** in PV cells following SE. Open circles indicate each individual value. Horizontal bars indicate mean value. Error bars indicate SEM (*p < 0.05 vs. control animals (Cont); *n* = 7, respectively). **(D)** Representative photos of active caspase-3 (red) positive mitochondria (Mito, green) in PV (blue) cells. Rectangles in merge panels (left) indicate the zoom areas for the high magnification photos (right). Bar = 5 (left panels) and 1.5 (right panels) μm.

**Figure 2:**
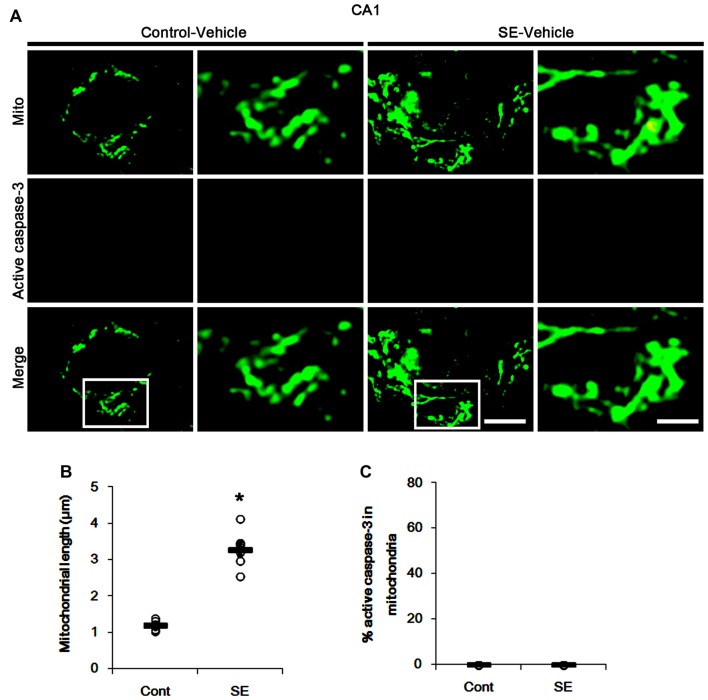
Aberrant mitochondrial fusion in CA1 neurons 3 days after SE. SE results in mitochondrial elongation and sphere formation in CA1 neurons of vehicle-treated animals. Active caspase-3 is not transported into mitochondria. **(A)** Representative photos of mitochondria (Mito, green) and active caspase-3 (red) signals in CA1 neurons. Rectangles in merge panels (left) indicate the zoom areas for the high magnification photos (right). Bar = 5 (left panels) and 1.5 (right panels) μm. **(B,C)** Quantification of the mitochondrial length **(B)** and the fraction of active caspase-3 positive mitochondria in total mitochondria **(C)** in CA1 neurons following SE. Open circles indicate each individual value. Horizontal bars indicate mean value. Error bars indicate SEM (*p < 0.05 vs. control animals (Cont); *n* = 7, respectively).

Since SE leads to PV cell apoptosis via excessive mitochondrial fission (Kim and Kang, 2017), we first investigated the relevance between mitochondrial dynamics and mitochondrial active caspase-3 transports. In control animals, mitochondrial length was ~1.25 μm in PV cells (Figures 1A,B). Active (cleaved) caspase-3 signal was not detected in mitochondria (Figures 1C,D). Six hours after SE, mitochondrial length in PV cells was reduced to ~0.29 μm (*p* < 0.05 vs. control; Figures 1A,B) and ~65% of total mitochondria showed active caspase-3 signals (*p* < 0.05 vs. control; Figures 1C,D). In contrast to PV cells, SE results in aberrant mitochondrial elongation in CA1 neurons (Kim et al., 2014; Hyun et al., 2016). Consistent with these reports, mitochondrial length was increased to ~3.29 μm in CA1 neurons 3 days after SE (*p* < 0.05 vs. control; Figures 2A,B). In addition, elongated mitochondria did not show active caspase-3 signals (Figures 2A,C). These findings indicate that SE-induced mitochondrial fragmentation may be accompanied by mitochondrial active caspase-3 translocation in PV cells, but not CA1 neurons.

### Mitochondrial Fragmentation Is Required for Active Caspase-3 Import to Mitochondria

Under physiological condition, mitochondrial fission is involved in a normal rate of cytochrome *c* release (Ishihara et al., 2009). In addition, mitochondrial fragmentation is an early apoptotic event, occurring before caspase activation (Suen et al., 2008). Thus, inhibition of mitochondrial fragmentation prevents release of cytochrome *c* and activation of caspase-mediated signaling pathway (Frank et al., 2001; Breckenridge et al., 2003; Lee et al., 2004; Germain et al., 2005; Barsoum et al., 2006). Indeed, we have reported that Mdivi-1 (a mitochondrial fission inhibitor) attenuates SE-induced PV cell apoptosis. Therefore, it is likely that mitochondrial fission may modulate mitochondrial active caspase-3 import leading to PV cell apoptosis. To confirm this hypothesis, we validated whether the regulations of mitochondrial dynamics influence mitochondrial active caspase-3 translocation in PV cells and CA1 neurons.

**Figure 3:**
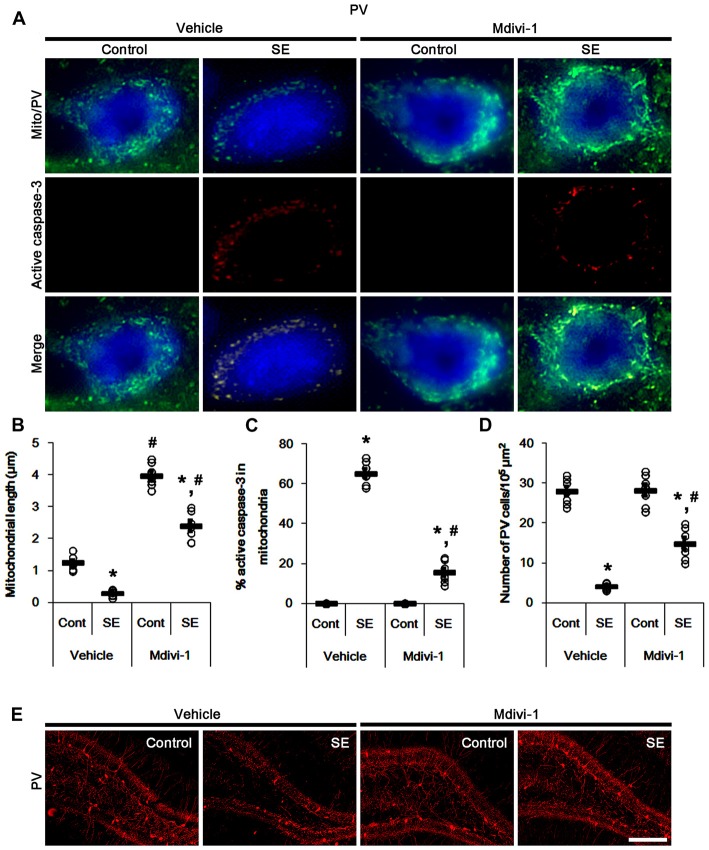
Effects of Mdivi-1 on mitochondrial dynamics, active caspase-3 translocation and PV cell loss following SE. Mdivi-1 attenuates mitochondrial fragmentation, active caspase-3 transport into mitochondria and PV cell loss induced by SE. **(A)** Representative photos of mitochondria (Mito, green) and active caspase-3 (red) in PV (blue) cells. Bar = 5 μm. **(B,C)** Quantification of the mitochondrial length **(B)** and the fraction of active caspase-3 positive mitochondria in total mitochondria **(C)** in PV cells following SE. Open circles indicate each individual value. Horizontal bars indicate mean value. Error bars indicate SEM (*,^#^*p* < 0.05 vs. control- (Cont) and vehicle-treated animals, respectively; *n* = 7, respectively). **(D)** Quantification of the number of PV cells 12 h after SE. Open circles indicate each individual value. Horizontal bars indicate mean value. Error bars indicate SEM (*,^#^*p* < 0.05 vs. control- (Cont) and vehicle-treated animals; *n* = 7, respectively). **(E)** Representative photos of PV cells 12 h after SE. Bar = 100 μm.

In control animals, Mdivi-1 effectively elongated mitochondrial length to >3 μm in PV cells. Following SE, it prevented mitochondrial fragmentation, active caspase-3 induction and its mitochondrial translocation in PV cells (*p* < 0.05 vs. vehicle; Figures 3A–C). Although Mdivi-1 did not induce PV cell viability under normal condition, it mitigated SE-induced PV cell loss 12 h after SE (*p* < 0.05 vs. vehicle; Figures 3D,E).

**Figure 4:**
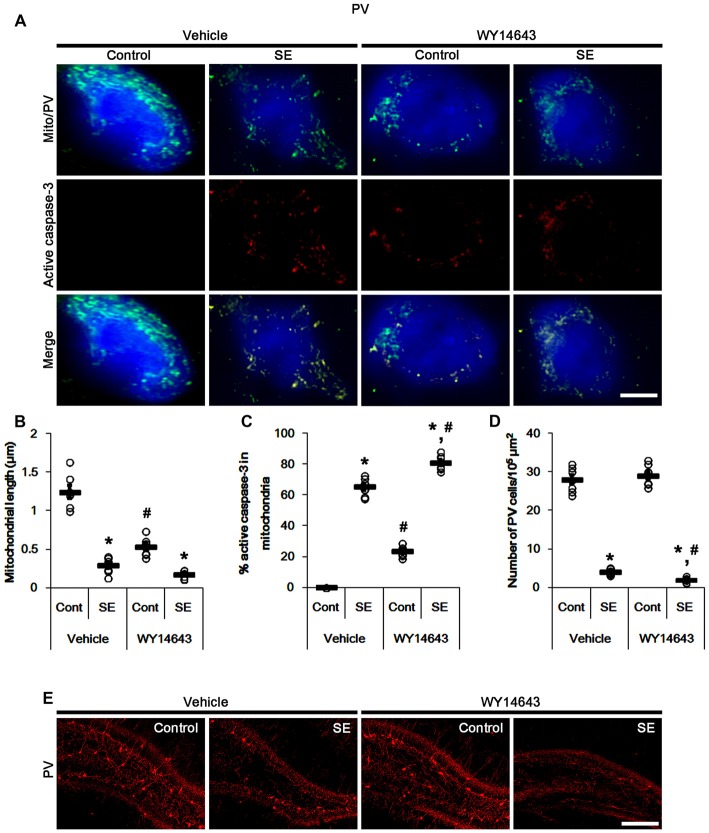
Effects of WY14643 on mitochondrial dynamics, active caspase-3 translocation and PV cell loss following SE. WY14643 deteriorates mitochondrial fragmentation, mitochondrial active caspase-3 transport and PV cell loss induced by SE. **(A)** Representative photos of mitochondria (green) and active caspase-3 (red) in PV (blue) cells. Bar = 5 μm. **(B,C)** Quantification of the mitochondrial length **(B)** and the fraction of active caspase-3 positive mitochondria in total mitochondria **(C)** in PV cells following SE. Open circles indicate each individual value. Horizontal bars indicate mean value. Error bars indicate SEM (*,^#^*p* < 0.05 vs. control-(Cont) and vehicle-treated animals, respectively; *n* = 7, respectively). **(D)** Quantification of the number of PV cells 12 h after SE. Open circles indicate each individual value. Horizontal bars indicate mean value. Error bars indicate SEM (*,^#^*p* < 0.05 vs. control-(Cont) and vehicle-treated animals, respectively; *n* = 7, respectively). **(E)** Representative photos of PV cells 12 h after SE. Bar = 100 μm.

In contrast to Mdivi-1, WY14643 (an activator of mitochondrial fission; Lundgren et al., 1990; Zolezzi et al., 2013; Kim et al., 2014) led to mitochondrial fragmentation in PV cells under normal condition. In addition, it resulted in mitochondrial active caspase-3 transports in ~24% of total mitochondria (*p* < 0.05 vs. vehicle; Figures 4A–C). Following SE, WY14643 facilitated active caspase-3 translocation into mitochondria, thus ~80% of total mitochondria showed active caspase-3 signals (*p* < 0.05 vs. vehicle; Figures 4A,C). Furthermore, WY14643 deteriorated SE-induced PV cell loss 12 h after SE (*p* < 0.05 vs. vehicle; Figures 4D,E), while it did not induce PV cell loss under normal condition.

**Figure 5:**
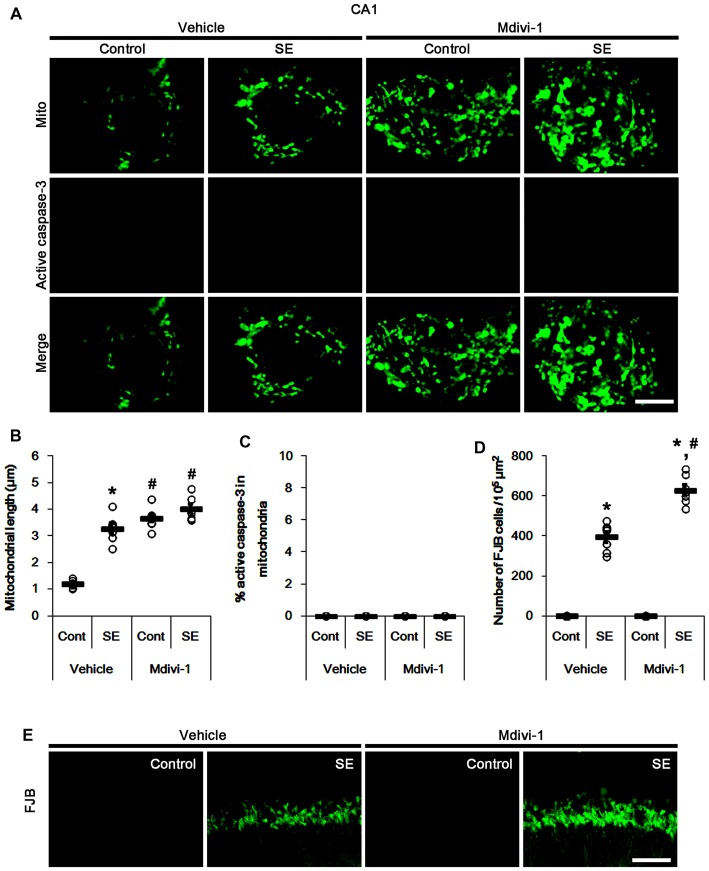
Effects of Mdivi-1 on mitochondrial dynamics, active caspase-3 translocation and CA1 neuronal death following SE. Mdivi-1 aggravates mitochondrial elongation and CA1 neuronal damage without altered mitochondrial active caspase-3 transport 3 days after SE. **(A)** Representative photos of mitochondria (Mito, green) and active caspase-3 (red) in CA1 neurons. Bar = 5 μm. **(B,C)** Quantification of the mitochondrial length **(B)** and the fraction of active caspase-3 positive mitochondria in total mitochondria **(C)** in CA1 neurons following SE. Open circles indicate each individual value. Horizontal bars indicate mean value. Error bars indicate SEM (*,^#^*p* < 0.05 vs. control-(Cont) and vehicle-treated animals, respectively; *n* = 7, respectively). **(D)** Quantification of the number of fluoro-jade B (FJB) positive CA1 neurons following SE. Open circles indicate each individual value. Horizontal bars indicate mean value. Error bars indicate SEM (*,^#^*p* < 0.05 vs. control- (Cont) and vehicle-treated animals, respectively; *n* = 7, respectively). **(E)** Representative photos of FJB positive CA1 neurons 3 days after SE. Bar = 100 μm.

Similar to PV cells, Mdivi-1 increased mitochondrial length in CA1 neurons of control- and post-SE animals (*p* < 0.05 vs. vehicle; Figures 5A,B). Mdivi-1 did not result in active caspase-3 induction and its mitochondrial translocation in CA1 neurons (Figures 5A,C). Although Mdivi-1 did not lead to CA1 neuronal death, it exacerbated SE-induced CA1 neuron degeneration 3 days after SE (*p* < 0.05 vs. vehicle; Figures 5D,E).

**Figure 6:**
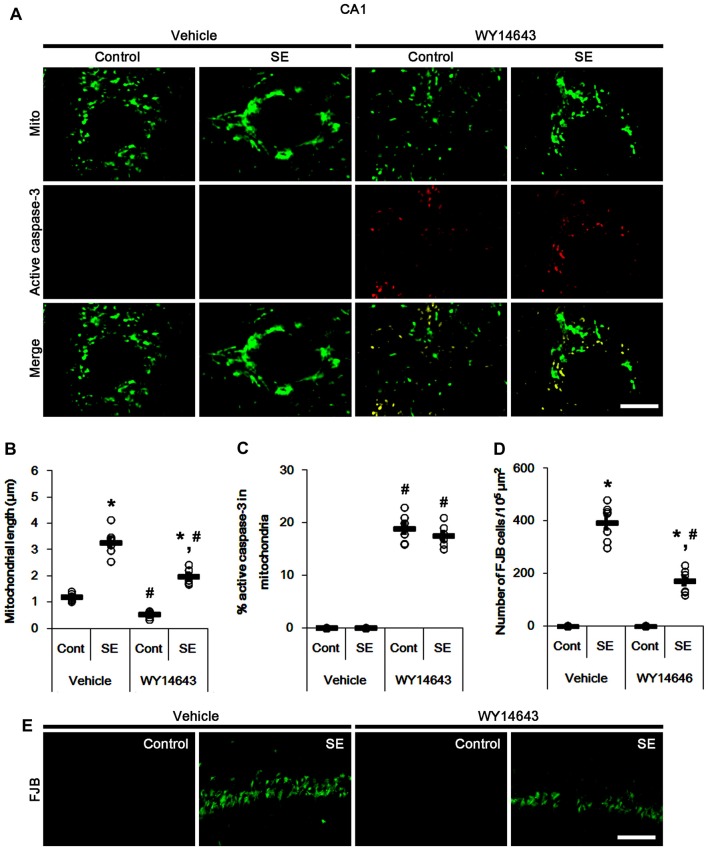
Effects of WY14643 on mitochondrial dynamics, active caspase-3 translocation and CA1 neuronal death following SE. WY14643 induces mitochondrial fission and mitochondrial translocation of active caspase-3 in control animals. WY14643 ameliorates mitochondrial elongation and CA1 neuronal damage without altering active caspase-3 transport 3 days after SE. **(A)** Representative photos of mitochondria (Mito, green) and active caspase-3 (red) in CA1 neurons. Bar = 5 μm. **(B,C)** Quantification of the mitochondrial length **(B)** and the fraction of active caspase-3 positive mitochondria in total mitochondria **(C)** in CA1 neurons following SE. Open circles indicate each individual value. Horizontal bars indicate mean value. Error bars indicate SEM (*,^#^*p* < 0.05 vs. control- (Cont) and vehicle-treated animals, respectively; *n* = 7, respectively). **(D)** Quantification of the number of FJB positive CA1 neurons following SE. Open circles indicate each individual value. Horizontal bars indicate mean value. Error bars indicate SEM (*,^#^*p* < 0.05 vs. control- (Cont) and vehicle-treated animals, respectively; *n* = 7, respectively). **(E)** Representative photos of FJB positive CA1 neurons 3 days after SE. Bar = 100 μm.

In control animals, WY14643 led mitochondrial fragmentation in CA1 neurons. It also induced mitochondrial active caspase-3 imports in ~19% of total mitochondria (*p* < 0.05 vs. vehicle; Figures 6A–C). However, WY14643 did not induce CA1 neuronal death under physiological condition (Figures 6D,E). Following SE, WY14643 attenuated mitochondrial elongation in CA1 neurons (*p* < 0.05 vs. vehicle; Figures 6A–C) and SE-induced CA1 neuronal death (*p* < 0.05 vs. vehicle; Figures 6D,E), although ~18% of total mitochondria showed active caspase-3 signals (Figures 6A–C). Taken together, these findings indicate that mitochondrial fission may play an important role in active caspase-3 induction and its mitochondrial imports in PV cells and CA1 neurons under normal conditions, although it did not lead to cell death. Furthermore, it is likely that active caspase-3 transports into mitochondrial may be relevant to PV cell loss, but not CA1 neuronal death following SE.

### Mitochondrial HMGB1 Import in CA1 Neurons, Not PV Cell, Induced by SE

**Figure 7:**
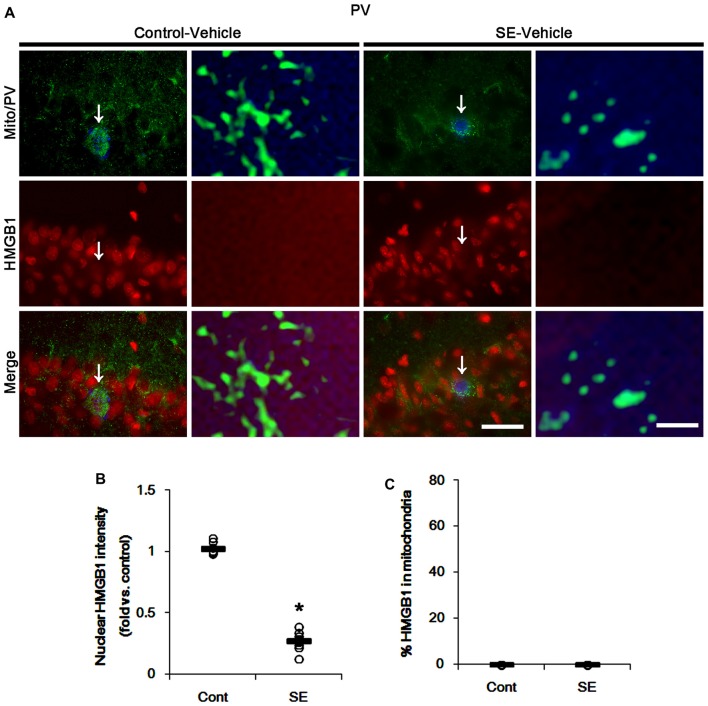
Mitochondrial fission and nuclear High mobility group box 1 (HMGB1) release in PV cells 6 h after SE. SE rapidly induces mitochondrial fragmentation and nuclear HMGB1 release in PV cells of vehicle-treated animals. However, HMGB1 is not imported into mitochondria. **(A)** Representative photos of mitochondria (Mito, green) and HMGB1 (red) signals in PV (blue) cells. Arrows in left panels indicate the zoom areas for the high magnification of right panels. Bar = 25 (left panels) and 1.5 (right panels) μm. **(B,C)** Quantification of the nuclear HMGB1 intensity **(B)** and the fraction of HMGB1 positive mitochondria in total mitochondria **(C)** in PV cells following SE. Open circles indicate each individual value. Horizontal bars indicate mean value. Error bars indicate SEM (*p < 0.05 vs. control animals (Cont); *n* = 7, respectively).

**Figure 8:**
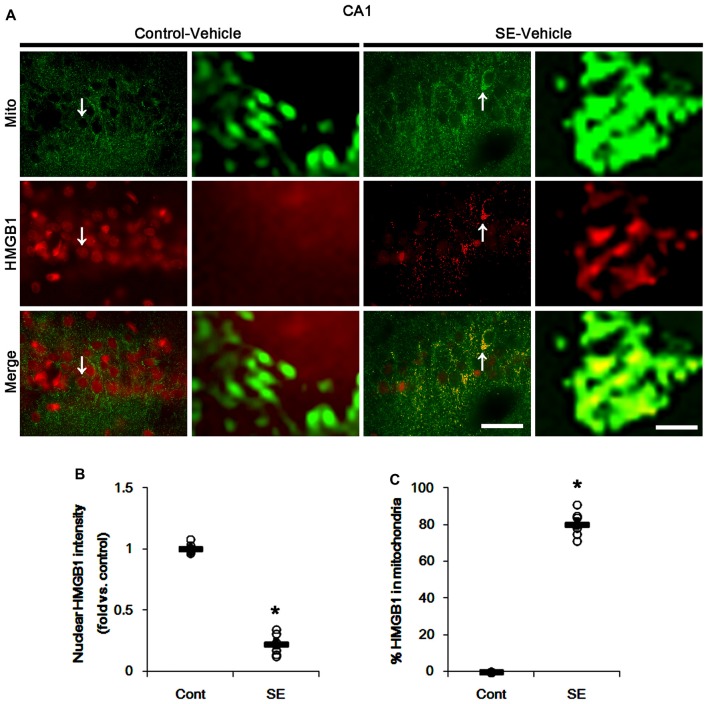
Mitochondrial fusion and HMGB1 transport to mitochondria in CA1 neurons 3 days after SE. SE results in mitochondrial elongation and HMGB1 translocation from nuclei to mitochondria in CA1 neurons of vehicle-treated animals. **(A)** Representative photos of mitochondria (Mito, green) and HMGB1 (red) signals in CA1 neurons. Arrows in left panels indicate the zoom areas for the high magnification of right panels. Bar = 25 (left panels) and 1.5 (right panels) μm. **(B,C)** Quantification of the nuclear HMGB1 intensity **(B)** and the fraction of HMGB1 positive mitochondria in total mitochondria **(B)** in CA1 neurons following SE. Open circles indicate each individual value. Horizontal bars indicate mean value. Error bars indicate SEM (*p < 0.05 vs. control animals (Cont); *n* = 7, respectively).

In various cells, nucleocytopalsmic HMGB1 release is observed during necrosis (Scaffidi et al., 2002; Faraco et al., 2007; Kim et al., 2014). Furthermore, translocation of HMGB1 to mitochondria may facilitate and deteriorate necrotic CA1 neuronal death (Hyun et al., 2016). Thus, we explored whether mitochondrial HMGB1 transports also influence SE-induced PV cell loss. In control animals, HMGB1 expression was restricted to the nuclei in PV cells. Six hours after SE, nuclear HMGB1 intensity was reduced in PV cells (*p* < 0.05 vs. control; Figures 7A,B). However, few fragmented mitochondria contained HMGB1 signals in PV cells (Figures 7A,C). In CA1 neurons, SE induced HMGB1 signal in elongated mitochondria with reduction in nuclear HMGB1 level (*p* < 0.05 vs. control; Figures 8A–C). These findings suggest that mitochondrial translocations of HMGB1 may be relevant to SE-induced necrotic CA1 neuronal death.

### SE-Induced Mitochondrial Elongation Accelerates Mitochondrial HMGB1 Translocation

**Figure 9:**
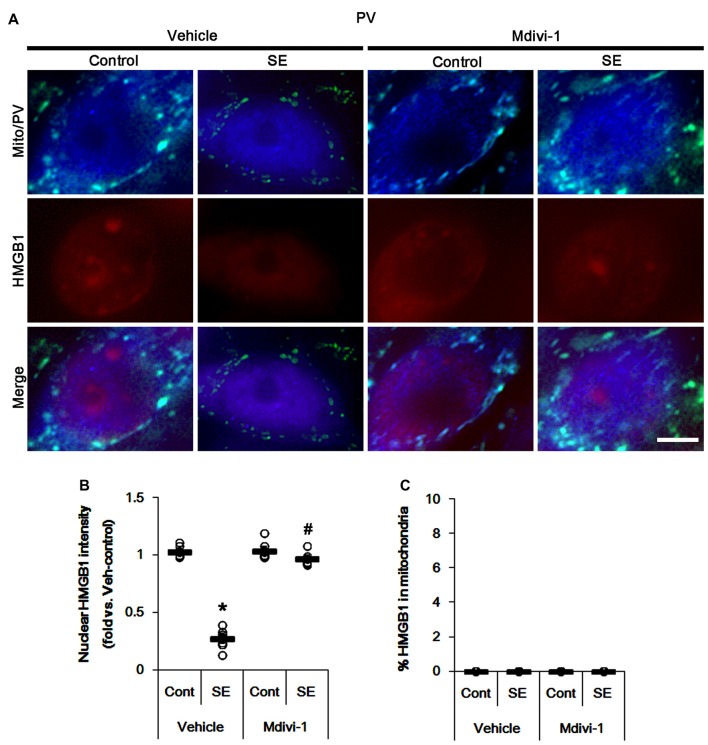
Effects of Mdivi-1 on mitochondrial dynamics and HMGB1 translocation in PV cells 6 h after SE. Mdivi-1 attenuates mitochondrial fragmentation and nuclear HMGB1 release 6 h after SE. Mdivi-1 does not induce HMGB1 translocation to mitochondria. **(A)** Representative photos of mitochondria (Mito, green) and HMGB1 (red) in PV (blue) cells. Bar = 5 μm. **(B,C)** Quantification of the nuclear HMGB1 intensity **(B)** and the fraction of HMGB1 positive mitochondria in total mitochondria **(C)** in PV cells 6 h after SE. Open circles indicate each individual value. Horizontal bars indicate mean value. Error bars indicate SEM (*,^#^*p* < 0.05 vs. control- (Cont) and vehicle-treated animals, respectively; *n* = 7, respectively).

**Figure 10:**
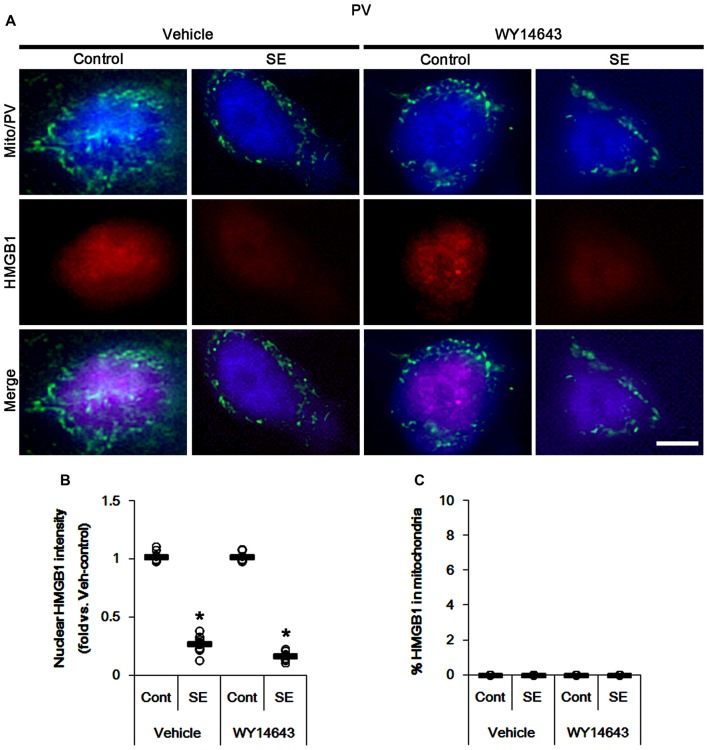
Effects of WY14643 on mitochondrial dynamics and HMGB1 translocation in PV cells 6 h after SE. WY14643 does not affect mitochondrial fragmentation and nuclear HMGB1 release following SE. In addition, WY14643 does not induce mitochondrial HMGB1 translocation. **(A)** Representative photos of mitochondria (Mito, green) and HMGB1 (red) in PV (blue) cells. Bar = 5 μm. **(B,C)** Quantification of the nuclear HMGB1 intensity **(B)** and the fraction of HMGB1 positive mitochondria in total mitochondria **(C)** in PV cells 6 h after SE. Open circles indicate each individual value. Horizontal bars indicate mean value. Error bars indicate SEM (*p < 0.05 vs. control animals (Cont); *n* = 7, respectively).

The remaining issue is whether mitochondrial dynamics also affect HMGB1 translocation to mitochondria in PV cells and CA1 neurons following SE. In the present study, Mdivi-1 and WY14643 did not lead to nuclear HMGB1 release in PV cells under physiological condition (Figures 9, 10). Mdivi-1 attenuated SE-induced nucleocytoplasmic HMGB1 translocation in PV cells (*p* < 0.05 vs. vehicle; Figures 9A,B). Mdivi-1 did not result in mitochondrial HMGB1 imports in PV cells of post-SE animals (Figures 9A,C). Although WY14643 could not abrogate nuclear HMGB1 release in PV cells (Figures 10A,B), it did not induce mitochondrial HMGB1 imports in PV cells of post-SE animals (Figures 10A,C).

**Figure 11:**
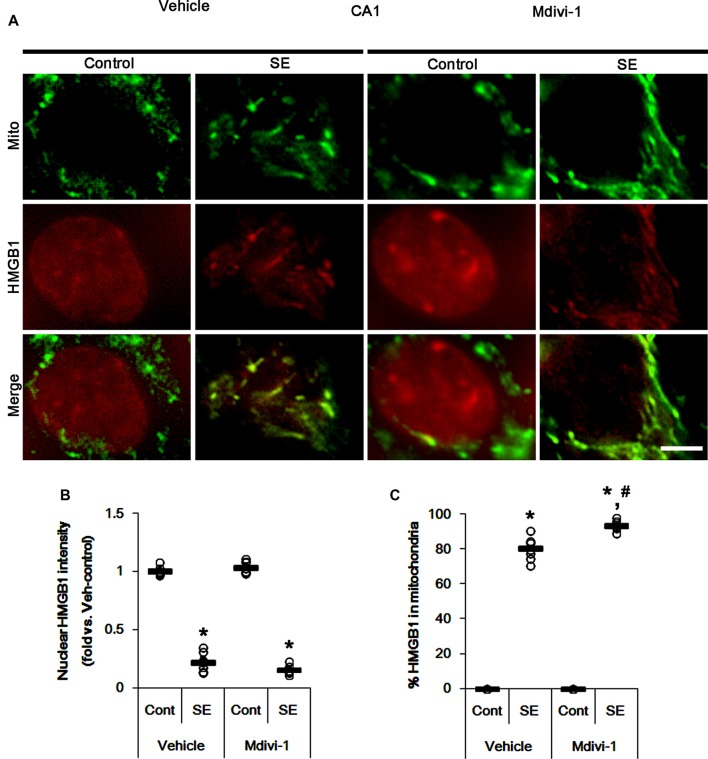
Effects of Mdivi-1 on mitochondrial dynamics and HMGB1 translocation in CA1 neurons 3 days after SE. Mdivi-1 aggravates mitochondrial elongation and mitochondrial HMGB1 translocation in CA1 neurons following SE. **(A)** Representative photos of mitochondria (Mito, green) and HMGB1 (red) in CA1 neurons following SE. Bar = 5 μm. **(B,C)** Quantification of the nuclear HMGB1 intensity **(B)** and the fraction of HMGB1 positive mitochondria in total mitochondria **(C)** in CA1 neurons 3 days after SE. Open circles indicate each individual value. Horizontal bars indicate mean value. Error bars indicate SEM (*,^#^*p* < 0.05 vs. control- (Cont) and vehicle-treated animals, respectively; *n* = 7, respectively).

**Figure 12:**
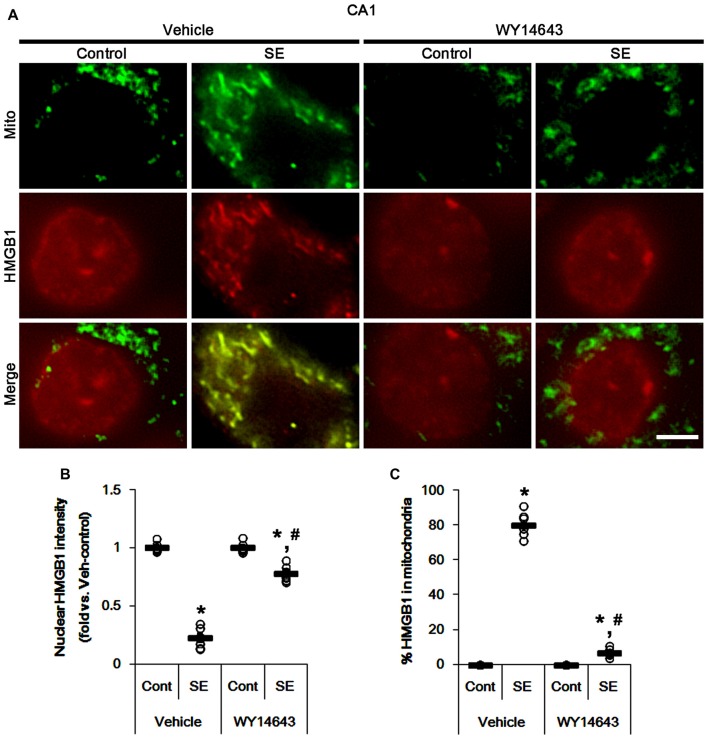
Effects of WY14643 on mitochondrial dynamics and HMGB1 translocation in CA1 neurons 3 days after SE. WY14643 mitigates mitochondrial elongation and mitochondrial HMGB1 translocation in CA1 neurons following SE. **(A)** Representative photos of mitochondria (Mito, green) and HMGB1 (red) in CA1 neurons following SE. Bar = 5 μm. **(B,C)** Quantification of the nuclear HMGB1 intensity **(B)** and the fraction of HMGB1 positive mitochondria in total mitochondria **(C)** in CA1 neurons 3 days after SE. Open circles indicate each individual value. Horizontal bars indicate mean value. Error bars indicate SEM (*,^#^*p* < 0.05 vs. control- (Cont) and vehicle-treated animals, respectively; *n* = 7, respectively).

Under normal conditions, both Mdivi-1 and WY14643 did not affect nuclear HMGB1 localization in CA1 neurons (Figures 11, 12). In post-SE animals, Mdivi-1 did not prevent nuclear HMGB1 export in CA1 neurons (Figures 11A,B). Furthermore, it enhanced SE-induced mitochondrial HMGB1 transport in CA1 neurons (*p* < 0.05 vs. vehicle; Figures 11A,C). However, WY14643 effectively attenuated nuclear HMGB1 release and its mitochondrial translocation (*p* < 0.05 vs. vehicle; Figures 12A–C). These findings indicate that mitochondrial dynamics may not participate in nuclear HMGB1 export and its mitochondrial translocation in both PV cells and CA1 neurons under normal conditions, but aberrant mitochondrial elongation may facilitate mitochondrial HMGB1 transport in CA1 neurons following SE.

## Discussion

Recently, we have reported that excessive mitochondrial fissions lead to apoptosis of PV cells in dentate gyrus following SE (Kim and Kang, 2017), while aberrant mitochondrial elongations evokes programed necrosis of CA1 neurons and dentate granule cells (Kim et al., 2014; Hyun et al., 2016; Ko and Kang, 2017). Consistent with these previous studies, the present data show that SE resulted in degenerations of PV cell and CA1 neurons accompanied by abnormal mitochondrial fission and fusion, respectively. Since mitochondrial dynamics are one of the important adaptive responses to the stressful stimuli (Chen and Chan, 2009; Rintoul and Reynolds, 2010), these findings indicate that the distinct impairment of mitochondrial dynamics may cause the different SE-induced cell death pattern between PV cells and CA1 neurons. However, the present study demonstrates that under physiological condition the inductions of mitochondrial fission or fusion by WY14643 and Mdivi-1 did not evoke neuronal death. Thus, it is likely that other factors may be involved in abnormal mitochondrial dynamics-mediated neuronal death under pathophysiological conditions.

PV cell loss is one of the most acute and dramatic events induced by SE (Soukupová et al., 2014; Kim and Kang, 2017). PV is one of the calcium-binding proteins, which is responsible for the fast-spiking capability of the GABAergic neurons, which participate in a rapid adaptation in response to repetitive stimuli. Thus, PV cell loss leads to uncontrolled discharges and further epileptogenic processes (Sloviter, 1991; Sloviter et al., 2003; Cammarota et al., 2013; Elgueta et al., 2015). SE-induced PV cell death is caspase-3 dependent apoptosis concomitant with mitochondrial fragmentation (Kang et al., 2006; Kim and Kang, 2017). Excessive mitochondrial fission by enhancing dynamin-related proteins 1 (DRP1)-serine 616 phosphorylation impairs mitochondrial function and increases susceptibility to apoptotic stimuli (Campello and Scorrano, 2010; Kim and Kang, 2017). This is because the released cytochrome *c* activates caspase-3 during mitochondrial fission (Frank et al., 2001; Breckenridge et al., 2003; Lee et al., 2004; Germain et al., 2005; Barsoum et al., 2006). Interestingly, the present study reveals the SE evoked massive active caspase-3 translocation into mitochondria of PV cells, accompanied by the reduced mitochondrial length. Furthermore, WY14643 enhanced mitochondrial active caspase-3 transport and deteriorated PV cell degeneration induced by SE, which were mitigated by Mdivi-1. Since active caspase-3 translocates into the mitochondria and disintegrates mitochondrial functions by degradation of mitochondrial constituents, especially in the late stage during apoptosis (Chandra and Tang, 2003), our findings suggest that preferential translocation of active caspase-3 into mitochondria may facilitate SE-induced apoptosis in PV cells, accompanied by excessive mitochondrial fission.

Unlike PV cells, SE induces abnormal mitochondrial elongation in CA1 neurons, in turn provokes programed necrosis independent of caspase-3 activity (Kim et al., 2014; Hyun et al., 2016). Impaired mitochondrial fission (aberrant mitochondrial elongation) exerts improper segregations of mitochondria and impaired mitochondrial transports, which result in neuronal death by reducing bioenergetics and respiratory function in peripheral sites of neurons (Parone et al., 2008; DuBoff et al., 2012; Kageyama et al., 2012; Kim et al., 2014). In the present study, SE led to aberrant mitochondrial elongation in CA1 neurons. Furthermore, Mdivi-1 increased the HMGB1 transport into mitochondria in CA1 neurons, and aggravated SE-induced CA1 cell loss. Together with data concerning mitochondrial active caspase-3 translocation in PV cells, our findings suggest that active caspase-3 and HMGB1 imports into mitochondria may be one of the regulatory factors in abnormal mitochondrial machinery-mediated neuronal death following SE.

Although nuclear HMGB1 release is an indicative of necrosis in various cells (Scaffidi et al., 2002; Faraco et al., 2007; Qiu et al., 2008), HMGB1 translocates into mitochondria and regulate their functions and reorganizations (Stumbo et al., 2008; Ito et al., 2014). Furthermore, translocation of HMGB1 into elongated mitochondria facilitates SE-induced CA1 neuronal death, while nuclear HMGB1 export could not affect mitochondrial dynamics (Hyun et al., 2016). In the present study, Mdivi-1 enhanced SE-induced mitochondrial HMGB1 transport in CA1 neurons, while was attenuated by WY14643. However, mitochondrial HMGB1 import was not observed in PV cell following SE, although nuclear HMGB1 release was detected. These findings indicate that the mitochondrial elongation may increase HMGB1 permeability into mitochondria. In addition, neither Mdivi-1 nor WY14643 resulted in mitochondrial HMGB1 transports in PV cells of control and post-SE animals. Therefore, our findings indicate that translocation of HMGB1 into mitochondria may be one of specific phenomena undergoing CA1 neuronal necrosis.

In the present study, under physiological condition WY14643 resulted in mitochondrial fissions and active caspase-3 translocations in ~24% and ~19% of total mitochondria of PV cells and CA1 neurons, respectively, although it did not induce the degenerations of these neurons. Since mitochondrial fission regulates a normal rate of cytochrome *c* release (Ishihara et al., 2009), these findings indicate that the rate of active caspase-3 into total mitochondria induced by WY14643 may be insufficient to evoke PV- and CA1 neuronal death under physiological condition. Indeed, WY14643 attenuated SE-induced CA1 neuronal death, in spite of active caspase-3 imports in ~18% of total mitochondria. Following SE, furthermore, degenerating PV cell showed active caspase-3 signals in ~65% of total mitochondria, which were increased to ~80% by WY14643. Taken together, our findings indicate that mitochondrial fission may be required for caspase-3 activation under normal and pathophysiological conditions, and suggest that SE-induced aberrant mitochondrial fusion in CA1 neurons may evoke necrosis rather than apoptosis due to inability of caspase-3 activation.

Why do PV cell and CA1 neurons show the distinct patterns of mitochondrial dynamics in response to pilocarpine-induced SE? We could not directly address this issue. Interestingly, the controversial effects of Mdivi-1 on SE-induced neuronal death would be considerable. Some reports demonstrate that Mdivi-1 attenuates neuronal loss after SE (Qiu et al., 2013; Xie et al., 2013), similar to the case of PV cells in the present study. However, the present study reveals that Mdivi-1 deteriorated SE-induced CA1 neuronal death. These discrepancies are resulted from the distinct methodology inducing SE: 1 h-lasting SE (Qiu et al., 2013; Xie et al., 2013) vs. 2 h-lasting models. Indeed, the differences in seizure activity lead to the distinct consequences on SE-induced neuronal death. Therefore, it is likely that the disparities in seizure susceptibility or firing rates during ictal stage may distinctly influence mitochondrial dynamics in different neuronal subpopulations. To validate this hypothesis, further studies are needed.

In conclusion, to the best of our knowledge, the present data provide the first evidence that SE-induced aberrant mitochondrial dynamics were involved in the mitochondrial translocations of active caspase-3 and HMGB1 in PV cells and CA1 neurons, respectively. Furthermore, these phenomena were closely relevant to the differential cell death patterns of PV cell and CA1 neurons in response to SE. Therefore, the identification of mitochondrial permeable molecules and their preferential events will be interesting and considerable topics to understand the cell death mechanisms relevant to impaired mitochondrial dynamics.

## Author Contributions

J-EK and T-CK designed the project. J-EK and T-CK performed the experiments described in the manuscript, analyzed the data and wrote the manuscript.

## Conflict of Interest Statement

The authors declare that the research was conducted in the absence of any commercial or financial relationships that could be construed as a potential conflict of interest.
